# Propane-1,3-diyl bis­(pyridine-3-carboxyl­ate)

**DOI:** 10.1107/S1600536810008810

**Published:** 2010-03-13

**Authors:** Iván Brito, Javier Vallejos, Michael Bolte, Matías López-Rodríguez

**Affiliations:** a Departamento de Química, Facultad de Ciencias Básicas, Universidad de Antofagasta, Casilla 170, Antofagasta, Chile; b Institut für Anorganische Chemie der Goethe-Universität Frankfurt, Max-von-Laue-Strasse 7, D-60438 Frankfurt am Main, Germany; c Instituto de Bio-Orgánica ’Antonio González’, Universidad de La Laguna, Astrofísico Francisco Sánchez N°2, La Laguna, Tenerife, Spain

## Abstract

The title compound, C_15_H_14_N_2_O_4_, has a *trans–gauche* [O/C/C/C–O/C/C/C] (TG) conformation. The angle between the planes of aromatic rings is 76.4 (3)°. The crystal structure is stabilized by van der Waals inter­actions and C—H⋯O hydrogen bonds. The crystal used was a non-merohedral twin with a fractional contribution of the minor component of 0.443 (5).

## Related literature

For conformation definitions, see: Carlucci *et al.* (2002 ▶). For applications of crystalline nanoporous coordination polymers, see Matsuda *et al.* (2005 ▶); Wu *et al.* (2005 ▶); Xiang *et al.* (2005 ▶).
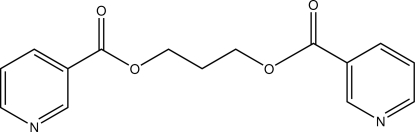


## Experimental

### 

#### Crystal data

C_15_H_14_N_2_O_4_
*M* *_r_* = 286.28
Triclinic, 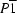

*a* = 4.4797 (11) Å
*b* = 10.911 (3) Å
*c* = 14.842 (4) Å
α = 104.41 (2)°
β = 95.90 (2)°
γ = 100.90 (2)°
*V* = 681.3 (3) Å^3^
*Z* = 2
Mo *K*α radiation
μ = 0.10 mm^−1^
*T* = 173 K
0.22 × 0.14 × 0.07 mm

#### Data collection

Stoe IPDS II two-circle diffractometer
8629 measured reflections
2558 independent reflections
1382 reflections with *I* > 2σ(*I*)
*R*_int_ = 0.119

#### Refinement

*R*[*F* ^2^ > 2σ(*F* ^2^)] = 0.109
*wR*(*F* ^2^) = 0.276
*S* = 1.49
2558 reflections
191 parameters
H-atom parameters constrained
Δρ_max_ = 0.43 e Å^−3^
Δρ_min_ = −0.46 e Å^−3^

### 

Data collection: *X-AREA* (Stoe & Cie, 2001 ▶); cell refinement: *X-AREA*; data reduction: *X-AREA*; program(s) used to solve structure: *SHELXS97* (Sheldrick, 2008 ▶); program(s) used to refine structure: *SHELXL97* (Sheldrick, 2008 ▶); molecular graphics: *XP* (Sheldrick, 2008 ▶); software used to prepare material for publication: *SHELXL97*.

## Supplementary Material

Crystal structure: contains datablocks I, global. DOI: 10.1107/S1600536810008810/om2324sup1.cif

Structure factors: contains datablocks I. DOI: 10.1107/S1600536810008810/om2324Isup2.hkl

Additional supplementary materials:  crystallographic information; 3D view; checkCIF report

## Figures and Tables

**Table 1:** Hydrogen-bond geometry (Å, °)

*D*—H⋯*A*	*D*—H	H⋯*A*	*D*⋯*A*	*D*—H⋯*A*
C3—H3*A*⋯O4^i^	0.99	2.49	3.341 (8)	144
C16—H16⋯O2^ii^	0.95	2.45	3.198 (10)	136
C24—H24⋯O2^iii^	0.95	2.45	3.218 (10)	138

Symmetry codes: (i) 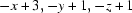; (ii) 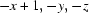; (iii) 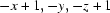.

**Fig. 1.**
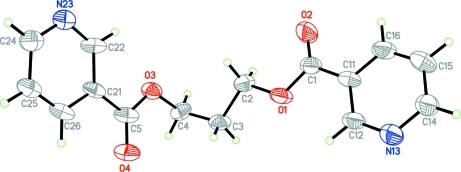
A view of the molecular structure with the atom-numbering scheme. Displacemenent ellipsoids are drawn at the 50% probability level and H atoms are shown as small spheres of arbitrary radii.

## supplementary crystallographic information

### Comment

In the past decade, crystalline nanoporous coordination polymers have been extensively studied for their potential applications in magnetism (Xiang *et al.*, 2005), catalysis (Wu *et al.*, 2005) and gas adsorption or separation (Matsuda *et al.*, 2005). The propanediyl group in the crystal structure can adopt four possible conformations: trans-trans (TT), trans-gauche (TG), gauche-gauche (GG), gauche-gauche' (GG') (Carlucci *et al.*, 2002). The propanediyl group in the title compound has a *trans-gauche* (TG)  [O1/C2/C3/C4 - O3/C4/C3/C2] conformation (Fig. 1). The angle between the planes of aromatics rings is 76.4 (3)°. The crystal structure is stabilized by van der Waals interactions and C—H···O hydrogen bonds (Table 1). To the best of our knowledge coordination polymer with this ligand still remain unknown.

### Experimental

Nicotinic acid (15 g, 0.122 mol) was stirred in SOCl_2 _(40 ml) in the presence of DMF (0.6 ml) at 60 °C for 12 h. Excess thionyl chloride was removed in vacuo. Dried propanediol (4.3 ml, 0.061 mol) was added. After the evolution of hydrogen chloride ended, the mixture was heated at 150 °C for 2 h. The mixture was dissolved in water, and NH_4_OH solution was added. After filtration, recrystallization in ethyl acetate gave colorless crystal. Yield 11.53 g (80 %). Analysis calculated for C_15_H_14_N_2_O_4_: C:62.9 , H:4.89 , N:9.68 ; found: C: 62.25, H: 4.68, N:9.52 . IR (KBr, cm^-1^): (C═O) 1715 s, (C═C) 1591 m, (Ar C—C, C═N) 1429 s,(C—O) 1277 m.

### Refinement

The crystal turned out to be a non-merohedral twin (twin law: -1 0 0/0 -0.476 -0.740/ 0 -1 0.478) with a fractional contribution of the minor component of 0.443 (5). H atoms were placed in idealized positions and treated as riding atoms with C—H distances in the range 0.95-0.99 Å and U_iso_(H) = 1.2U_eq_(C). The material was difficult to obtain in a suitable crystalline form.

### Crystal data

**Table:**

C_15_H_14_N_2_O_4_	*Z* = 2
*M**_r_* = 286.28	*F*(000) = 300
Triclinic, *P*1	*D*_x_ = 1.395 Mg m^−^^3^
Hall symbol: -P 1	Mo *K*α radiation, λ = 0.71073 Å
*a* = 4.4797 (11) Å	Cell parameters from 3924 reflections
*b* = 10.911 (3) Å	θ = 3.9–25.8°
*c* = 14.842 (4) Å	µ = 0.10 mm^−^^1^
α = 104.41 (2)°	*T* = 173 K
β = 95.90 (2)°	Plate, colourless
γ = 100.90 (2)°	0.22 × 0.14 × 0.07 mm
*V* = 681.3 (3) Å^3^	

### Data collection

**Table:**

Stoe IPDS II two-circle diffractometer	1382 reflections with *I* > 2σ(*I*)
Radiation source: fine-focus sealed tube	*R*_int_ = 0.119
graphite	θ_max_ = 26.0°, θ_min_ = 3.8°
ω scans	*h* = −5→5
8629 measured reflections	*k* = −13→12
2558 independent reflections	*l* = −12→17

### Refinement

**Table:**

Refinement on *F*^2^	Primary atom site location: structure-invariant direct methods
Least-squares matrix: full	Secondary atom site location: difference Fourier map
*R*[*F*^2^ > 2σ(*F*^2^)] = 0.109	Hydrogen site location: inferred from neighbouring sites
*wR*(*F*^2^) = 0.276	H-atom parameters constrained
*S* = 1.49	*w* = 1/[σ^2^(*F*_o_^2^) + (0.090*P*)^2^] where *P* = (*F*_o_^2^ + 2*F*_c_^2^)/3
2558 reflections	(Δ/σ)_max_ < 0.001
191 parameters	Δρ_max_ = 0.43 e Å^−^^3^
0 restraints	Δρ_min_ = −0.46 e Å^−^^3^

### Special details

**Table:**

Geometry. All esds (except the esd in the dihedral angle between two l.s. planes) are estimated using the full covariance matrix. The cell esds are taken into account individually in the estimation of esds in distances, angles and torsion angles; correlations between esds in cell parameters are only used when they are defined by crystal symmetry. An approximate (isotropic) treatment of cell esds is used for estimating esds involving l.s. planes.
Refinement. Refinement of *F*^2^ against ALL reflections. The weighted *R*-factor wR and goodness of fit *S* are based on *F*^2^, conventional *R*-factors *R* are based on *F*, with *F* set to zero for negative *F*^2^. The threshold expression of *F*^2^ > σ(*F*^2^) is used only for calculating *R*-factors(gt) etc. and is not relevant to the choice of reflections for refinement. *R*-factors based on *F*^2^ are statistically about twice as large as those based on *F*, and *R*- factors based on ALL data will be even larger.

### Fractional atomic coordinates and isotropic or equivalent isotropic displacement parameters (Å^2^)

**Table:**

	*x*	*y*	*z*	*U*_iso_*/*U*_eq_	
O1	0.8187 (10)	0.2723 (4)	0.2445 (4)	0.0458 (12)	
O2	0.6697 (13)	0.0749 (5)	0.1430 (4)	0.0637 (16)	
O3	1.1653 (9)	0.2272 (4)	0.5109 (3)	0.0405 (11)	
O4	1.2061 (11)	0.4252 (4)	0.6081 (4)	0.0540 (14)	
C1	0.6560 (14)	0.1865 (6)	0.1644 (5)	0.0434 (17)	
C2	1.0002 (15)	0.2161 (6)	0.3054 (5)	0.0434 (16)	
H2A	1.1296	0.1648	0.2688	0.052*	
H2B	0.8629	0.1587	0.3334	0.052*	
C3	1.2023 (14)	0.3317 (6)	0.3827 (5)	0.0448 (17)	
H3A	1.3515	0.3838	0.3543	0.054*	
H3B	1.0718	0.3881	0.4135	0.054*	
C4	1.3748 (14)	0.2826 (7)	0.4557 (5)	0.0445 (17)	
H4A	1.4795	0.2160	0.4234	0.053*	
H4B	1.5339	0.3554	0.4981	0.053*	
C5	1.0994 (13)	0.3111 (6)	0.5848 (5)	0.0439 (17)	
C11	0.4701 (14)	0.2473 (6)	0.1081 (5)	0.0420 (16)	
C12	0.4516 (15)	0.3777 (6)	0.1359 (5)	0.0451 (17)	
H12	0.5646	0.4308	0.1948	0.054*	
N13	0.2835 (15)	0.4308 (6)	0.0838 (5)	0.0568 (17)	
C14	0.1172 (16)	0.3544 (7)	0.0009 (6)	0.0538 (19)	
H14	−0.0070	0.3904	−0.0367	0.065*	
C15	0.1253 (17)	0.2229 (7)	−0.0306 (6)	0.055 (2)	
H15	0.0121	0.1712	−0.0899	0.066*	
C16	0.2940 (17)	0.1699 (7)	0.0235 (6)	0.0516 (19)	
H16	0.2923	0.0800	0.0037	0.062*	
C21	0.8829 (13)	0.2446 (6)	0.6378 (5)	0.0386 (15)	
C22	0.7613 (14)	0.1126 (6)	0.6107 (6)	0.0472 (18)	
H22	0.8192	0.0626	0.5560	0.057*	
N23	0.5693 (13)	0.0501 (6)	0.6553 (4)	0.0505 (16)	
C24	0.4879 (17)	0.1254 (7)	0.7329 (6)	0.0526 (19)	
H24	0.3486	0.0843	0.7661	0.063*	
C25	0.5981 (16)	0.2594 (6)	0.7660 (5)	0.0470 (17)	
H25	0.5369	0.3086	0.8205	0.056*	
C26	0.7973 (14)	0.3177 (6)	0.7173 (5)	0.0446 (17)	
H26	0.8772	0.4089	0.7381	0.054*	

### Atomic displacement parameters (Å^2^)

**Table:**

	*U*^11^	*U*^22^	*U*^33^	*U*^12^	*U*^13^	*U*^23^
O1	0.045 (2)	0.027 (2)	0.054 (3)	0.0010 (19)	−0.005 (2)	0.001 (2)
O2	0.095 (4)	0.041 (3)	0.047 (3)	0.020 (3)	−0.005 (3)	−0.001 (2)
O3	0.036 (2)	0.035 (2)	0.043 (3)	−0.0023 (18)	0.005 (2)	0.004 (2)
O4	0.051 (3)	0.032 (3)	0.066 (4)	−0.004 (2)	0.006 (2)	0.003 (2)
C1	0.043 (3)	0.033 (4)	0.048 (5)	0.002 (3)	0.003 (3)	0.008 (3)
C2	0.044 (3)	0.033 (3)	0.053 (4)	0.006 (3)	0.006 (3)	0.014 (3)
C3	0.037 (3)	0.031 (3)	0.056 (4)	−0.008 (3)	−0.001 (3)	0.009 (3)
C4	0.041 (3)	0.040 (4)	0.054 (5)	0.002 (3)	0.015 (3)	0.020 (3)
C5	0.034 (3)	0.027 (3)	0.062 (5)	0.002 (3)	−0.001 (3)	0.002 (3)
C11	0.046 (3)	0.025 (3)	0.046 (4)	0.004 (3)	0.005 (3)	−0.002 (3)
C12	0.053 (4)	0.027 (3)	0.048 (5)	0.007 (3)	0.003 (3)	0.001 (3)
N13	0.072 (4)	0.039 (3)	0.051 (4)	0.013 (3)	−0.006 (3)	0.003 (3)
C14	0.057 (4)	0.041 (4)	0.057 (5)	0.012 (3)	−0.009 (4)	0.008 (4)
C15	0.059 (4)	0.043 (4)	0.049 (5)	0.008 (3)	0.007 (4)	−0.010 (3)
C16	0.066 (4)	0.030 (3)	0.051 (5)	0.006 (3)	0.009 (4)	0.001 (3)
C21	0.031 (3)	0.029 (3)	0.044 (4)	0.001 (2)	−0.007 (3)	−0.002 (3)
C22	0.045 (3)	0.024 (3)	0.063 (5)	0.001 (3)	0.003 (3)	0.003 (3)
N23	0.054 (3)	0.041 (3)	0.048 (4)	−0.002 (3)	0.004 (3)	0.008 (3)
C24	0.058 (4)	0.040 (4)	0.055 (5)	0.004 (3)	0.011 (4)	0.010 (4)
C25	0.050 (4)	0.034 (3)	0.047 (4)	0.006 (3)	−0.002 (3)	−0.002 (3)
C26	0.042 (3)	0.028 (3)	0.051 (5)	0.004 (3)	0.000 (3)	−0.008 (3)

### Geometric parameters (Å, °)

**Table:**

O1—C1	1.353 (8)	C12—N13	1.329 (10)
O1—C2	1.472 (8)	C12—H12	0.9500
O2—C1	1.194 (8)	N13—C14	1.353 (10)
O3—C5	1.340 (8)	C14—C15	1.402 (10)
O3—C4	1.452 (7)	C14—H14	0.9500
O4—C5	1.193 (8)	C15—C16	1.349 (12)
C1—C11	1.475 (10)	C15—H15	0.9500
C2—C3	1.537 (9)	C16—H16	0.9500
C2—H2A	0.9900	C21—C22	1.379 (8)
C2—H2B	0.9900	C21—C26	1.384 (9)
C3—C4	1.528 (10)	C22—N23	1.328 (9)
C3—H3A	0.9900	C22—H22	0.9500
C3—H3B	0.9900	N23—C24	1.364 (10)
C4—H4A	0.9900	C24—C25	1.393 (9)
C4—H4B	0.9900	C24—H24	0.9500
C5—C21	1.504 (10)	C25—C26	1.368 (10)
C11—C16	1.389 (10)	C25—H25	0.9500
C11—C12	1.400 (8)	C26—H26	0.9500
			
C1—O1—C2	114.8 (5)	N13—C12—C11	123.2 (7)
C5—O3—C4	116.2 (5)	N13—C12—H12	118.4
O2—C1—O1	122.4 (6)	C11—C12—H12	118.4
O2—C1—C11	125.2 (6)	C12—N13—C14	118.4 (7)
O1—C1—C11	112.4 (6)	N13—C14—C15	121.1 (7)
O1—C2—C3	105.9 (5)	N13—C14—H14	119.5
O1—C2—H2A	110.6	C15—C14—H14	119.5
C3—C2—H2A	110.6	C16—C15—C14	119.9 (7)
O1—C2—H2B	110.6	C16—C15—H15	120.0
C3—C2—H2B	110.6	C14—C15—H15	120.0
H2A—C2—H2B	108.7	C15—C16—C11	119.8 (7)
C4—C3—C2	109.8 (6)	C15—C16—H16	120.1
C4—C3—H3A	109.7	C11—C16—H16	120.1
C2—C3—H3A	109.7	C22—C21—C26	117.6 (7)
C4—C3—H3B	109.7	C22—C21—C5	123.1 (6)
C2—C3—H3B	109.7	C26—C21—C5	119.3 (6)
H3A—C3—H3B	108.2	N23—C22—C21	125.0 (7)
O3—C4—C3	111.0 (5)	N23—C22—H22	117.5
O3—C4—H4A	109.4	C21—C22—H22	117.5
C3—C4—H4A	109.4	C22—N23—C24	115.8 (6)
O3—C4—H4B	109.4	N23—C24—C25	123.6 (7)
C3—C4—H4B	109.4	N23—C24—H24	118.2
H4A—C4—H4B	108.0	C25—C24—H24	118.2
O4—C5—O3	124.4 (6)	C26—C25—C24	117.7 (7)
O4—C5—C21	123.4 (7)	C26—C25—H25	121.2
O3—C5—C21	112.2 (5)	C24—C25—H25	121.2
C16—C11—C12	117.6 (7)	C25—C26—C21	120.3 (6)
C16—C11—C1	118.2 (6)	C25—C26—H26	119.8
C12—C11—C1	124.1 (6)	C21—C26—H26	119.8
			
C2—O1—C1—O2	−3.1 (9)	N13—C14—C15—C16	1.9 (12)
C2—O1—C1—C11	177.9 (5)	C14—C15—C16—C11	−2.9 (11)
C1—O1—C2—C3	171.4 (5)	C12—C11—C16—C15	3.2 (10)
O1—C2—C3—C4	173.9 (5)	C1—C11—C16—C15	−178.9 (7)
C5—O3—C4—C3	−84.3 (7)	O4—C5—C21—C22	−179.8 (7)
C2—C3—C4—O3	−70.1 (7)	O3—C5—C21—C22	−1.8 (9)
C4—O3—C5—O4	−1.7 (10)	O4—C5—C21—C26	0.8 (10)
C4—O3—C5—C21	−179.6 (6)	O3—C5—C21—C26	178.7 (6)
O2—C1—C11—C16	0.4 (11)	C26—C21—C22—N23	−0.4 (11)
O1—C1—C11—C16	179.4 (6)	C5—C21—C22—N23	−179.9 (7)
O2—C1—C11—C12	178.2 (7)	C21—C22—N23—C24	0.9 (11)
O1—C1—C11—C12	−2.8 (9)	C22—N23—C24—C25	−0.9 (11)
C16—C11—C12—N13	−2.5 (10)	N23—C24—C25—C26	0.3 (12)
C1—C11—C12—N13	179.7 (7)	C24—C25—C26—C21	0.2 (10)
C11—C12—N13—C14	1.4 (11)	C22—C21—C26—C25	−0.2 (10)
C12—N13—C14—C15	−1.1 (11)	C5—C21—C26—C25	179.3 (7)

### Hydrogen-bond geometry (Å, °)

**Table:**

*D*—H···*A*	*D*—H	H···*A*	*D*···*A*	*D*—H···*A*
C3—H3A···O4^i^	0.99	2.49	3.341 (8)	144
C16—H16···O2^ii^	0.95	2.45	3.198 (10)	136
C24—H24···O2^iii^	0.95	2.45	3.218 (10)	138

Symmetry codes: (i) −*x*+3, −*y*+1, −*z*+1; (ii) −*x*+1, −*y*, −*z*; (iii) −*x*+1, −*y*, −*z*+1.
